# Cyclosporine A Regulates Influenza A Virus-induced Macrophages Polarization and Inflammatory Responses by Targeting Cyclophilin A

**DOI:** 10.3389/fimmu.2022.861292

**Published:** 2022-05-25

**Authors:** Xiaoyuan Bai, Wenxian Yang, Heqiao Li, Yuna Zhao, Wenhui Fan, He Zhang, Wenjun Liu, Lei Sun

**Affiliations:** ^1^ CAS Key Laboratory of Pathogenic Microbiology and Immunology, Institute of Microbiology, Chinese Academy of Sciences (CAS), Beijing, China; ^2^ Institute of Infectious Diseases, Shenzhen Bay Laboratory, Shenzhen, China; ^3^ Savaid Medical School, University of Chinese Academy of Sciences, Beijing, China; ^4^ State Key Laboratory for Conservation and Utilization of Subtropical Agro-Bioresources & Laboratory of Animal Infectious Diseases, College of Animal Sciences and Veterinary Medicine, Guangxi University, Nanning, China

**Keywords:** influenza A virus, macrophages polarization, cyclosporine A, cyclophilin A, inflammation

## Abstract

Cyclosporine A (CsA) is an immunosuppressive drug that suppresses T cell responses and is broadly used in transplantation. Its immunosuppressive action is closely linked to its binding of cyclophilin A (CypA), which widely distributed in different cell types. CsA also regulates the functions of innate immune cells, but the mechanism remains elusive. Here, we investigate the role of CsA in regulating macrophages polarization in influenza A virus-infected mice and mouse bone marrow-derived macrophages. CsA downregulates pro-inflammatory cytokines expression and upregulates anti-inflammatory cytokines expression. Mechanically, CsA decreases the polarization of macrophages into pro-inflammatory M1 phenotype and increases the polarization of macrophages into anti-inflammatory M2 phenotype. Further studies show that CsA regulates macrophages polarization-associated IFN-γ/STAT1 and IL-4/STAT6 signaling pathways. Meanwhile, all these roles of CsA are eliminated when CypA is absent, suggesting that CsA regulates macrophages polarization and inflammatory responses depend on its binding to CypA. Collectively, these results reveal a crucial mechanism of CsA in attenuating IAV-induced inflammatory responses by a switch in macrophages polarization.

## Introduction

Historically, influenza A virus is the most common cause of lower respiratory tract infection that can result in acute pneumonia, which has a high mortality rate during epidemics and especially during pandemics (1, 2). At the early stage of virus infection, IAV triggers the innate immune responses, recruits immune cells (macrophages, neutrophils and dendritic cells) to lungs (3), and excess inflammatory cytokines are secreted from these activated cells, such as TNF-α, IL6, and IL1β, thereby leading to acute pneumonia (4, 5). Hence, maintaining inflammatory responses homeostasis is effective for the treatment of influenza virus-induced pneumonia.

Cyclosproine A (CsA) is a cyclic 11-amino-acid peptide, which produced by the fungus *Tolypocladium inflatum* (6). It binds to cyclophilin A (CypA, encoded by *PPIA*) firstly, which is the major intracellular receptor for CsA, and then forms a ternary complex with calcineurin, leading to the inhibition of calcineurin and nuclear factor of activated T cells (NFAT), which negatively regulates the expression of T cell related cytokines, and thereby suppresses the activation of T cells (7). Additionally, as the prototypical inhibitor of CypA, CsA blocks the isomerase activity of CypA with relatively high affinity (8). CsA was firstly marketed in the mid-1980s. Based on its immunosuppressive activity, CsA was widely used in transplantation for organ transplant patients to reduce the risk of organ rejection and effective in alleviating autoimmune diseases, such as spontaneous urticarial (9), rheumatism (10) and systemic lupus erythematosus (11). In addition, substantial evidence shows that CsA is helpful to relieve acute inflammatory diseases, such as sepsis (12–14) and endotoxemia (15), due to its important role in regulating the functions of innate immune cells (such as monocytes/macrophages, dendritic cells, and neutrophils), vascular activity, the release of cytokines, mitochondrial dysfunction, and apoptosis (16). Additionally, CsA relieves IAV-induced immunopathological damage (17). However, how CsA regulate inflammatory responses remains less understood.

Macrophages play vital roles in virus-triggered inflammatory responses. Upon IAV infection, activated macrophages polarize into M1 macrophages (classical activated macrophage) or M2 macrophages (alternatively activated macrophage) (18). M1 and M2 macrophages have distinct functions in the regulation of the inflammatory responses (19). M1 macrophages produce pro-inflammatory cytokines and play a role in virus clearance, but excess inflammation is harmful to the host (20). On the contrary, M2 macrophages inhibit excess inflammatory responses and promote tissue repair (20, 21). Although it has been reported that mesenchymal stem cells applied in combination with CsA may regulate the polarization of macrophages, thereby inhibiting the inflammatory responses in allogeneic skin transplantation model (22), the roles of CsA in influenza virus-induced macrophages polarization is still unknown.

In the present study, we discover that CsA regulates IAV-induced macrophages polarization by targeting CypA and inhibits the inflammatory responses, indicating that CsA could be a potential drug for the therapy of virus-induced acute inflammatory responses and tissue damage.

## Results

### CsA Suppresses IAV-Triggered Inflammatory Responses

**Figure 1:**
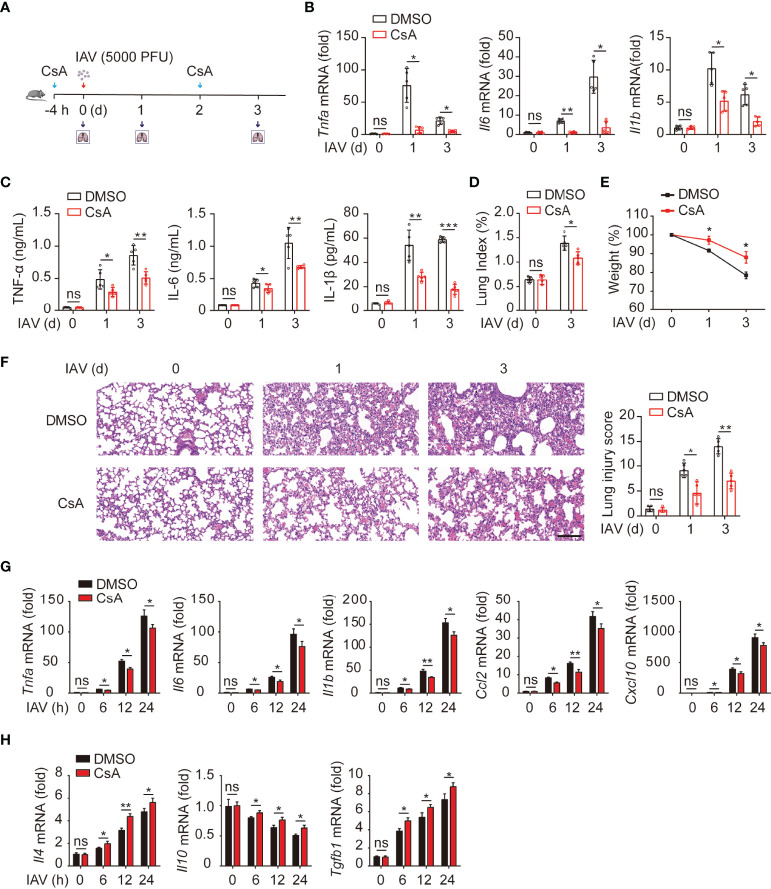
Cyclosproine A (CsA) suppresses IAV-triggered inflammatory responses both *in vivo* and *in vitro.* **(A)** Mice were injected intraperitoneally with CsA (20 mg/kg/48 h) or DMSO for 4 hours (h) before infected intranasally with 5000 PFU of influenza A/WSN/33 (H1N1) (n = 5). The same treatment of CsA was administered at day 2 post infection. **(B)** Real-time PCR analysis of *Tnfa*, *Il6*, and *Il1b* mRNA in mouse lungs at various time points. **(C)** ELISA analysis of TNF-α, IL-6, and IL-1β in mouse bronchoalveolar lavage fluid (BALF). **(D)** The lung index (100× lung/body weight) was calculated at day 0 and 3 post infection. **(E)** The body weight of mice was monitored at day 0, 1, and 3 d after IAV infection. **(F)** Hematoxylin and eosin (H & E) staining of mouse lungs (left). The severity of the lung injury was analyzed in a blinded manner. Scale bar, 100 μm. **(G)** Real-time PCR analysis of inflammatory cytokines and chemokines mRNA in BMDMs pretreated with CsA (5 μM) or DMSO for 2 h, followed by infection with IAV (multiplicity of infection [MOI] = 1) for various times. **(H)** Real-time PCR analysis of anti-inflammatory cytokines mRNA in BMDMs treated as described in **(G)**. Data shown are representative of three independent experiments. Data are presented as the mean ± SD. ns, not significant; *p < 0.05; **p < 0.01; ***p < 0.001 (unpaired, two-tailed Student’s t-test).

It has been documented that CsA reduces the immunopathological damage and facilitates the survival of IAV-infected mice (17). We further investigated the role of CsA in IAV-induced inflammatory responses in mouse lungs. An influenza A/WSN/33 (H1N1)-infected mouse model was established **(** **Figure 1A** **)**. Mice were injected intraperitoneally with CsA for 4 hours (h) before infected intranasally with IAV. The solution with 2% DMSO was as a negative control. The same treatment of CsA was administered at day 2 post infection. The mRNA and protein levels of pro-inflammation cytokines were determined firstly. CsA reduced the mRNA levels of TNF-α, IL-6, and IL-1β in mouse lungs **(** **Figure 1B** **)**. Meanwhile, similar results were observed in the levels of these cytokines in bronchoalveolar lavage fluid (BALF) **(** **Figure 1C** **)**. Then the effects of CsA on the pathogenicity of IAV-infected mice were investigated. After IAV infection, the lung indices (100× lung/body weight) of CsA-pretreated mice were lower than those of control mice **(** **Figure 1D** **)**. Consistently, a slower weight loss of CsA-pretreated mice was observed compared with that of control mice **(** **Figure 1E** **)**. In addition, CsA alleviated lung consolidation with less infiltrated inflammatory cells **(** **Figure 1F** **)**. These results indicate that CsA inhibits IAV-induced inflammatory cytokines production and lung injury *in vivo*.

Next, the role of CsA in IAV-induced inflammatory responses was examined in bone marrow-derived macrophages (BMDMs) from mice. The results of real-time PCR revealed that CsA inhibited the mRNA levels of TNF-α, IL6, IL1β, CCL2, and CXCL10 in IAV-infected BMDMs **(** **Figure 1G** **)**. In contrast, the expression of anti-inflammatory cytokines (IL4 and TGF-β) was up-regulated in CsA-treated BMDMs upon IAV infection compared with that in DMSO-treated BMDMs **(** **Figure 1H** **)**. Taken together, these results indicate that CsA suppresses IAV-triggered inflammatory responses both *in vivo* and *in vitro*.

### CsA Regulates Macrophages Polarization

**Figure 2:**
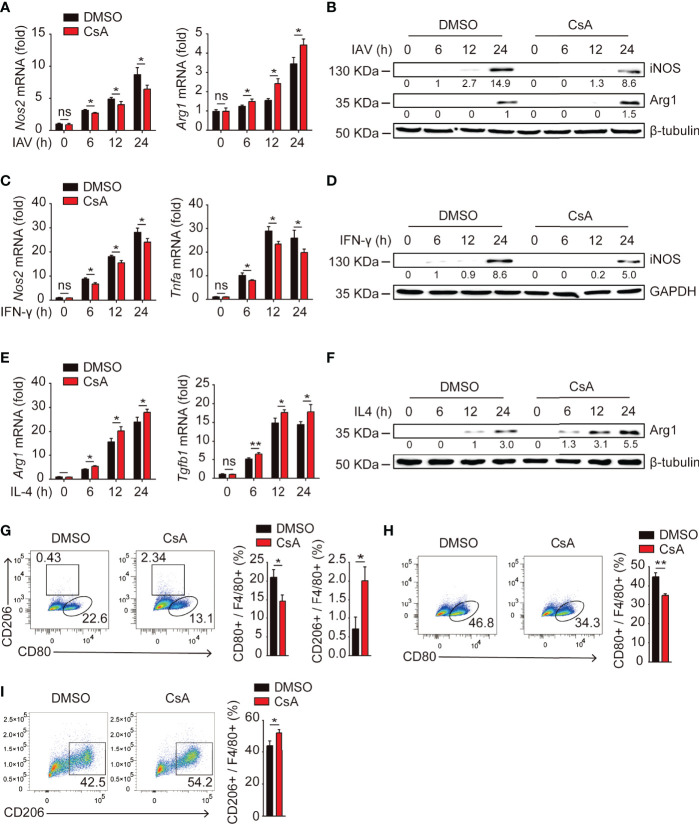
CsA regulates macrophages polarization. **(A)** Real-time PCR analysis of *Nos2* and *Arg1* mRNA in BMDMs pretreated with CsA (5 μM) or 0.1% DMSO for 2 h, followed by infection with IAV (MOI = 1) for various times. **(B)** Western blotting analysis of iNOS and Arg1 protein in BMDMs treated in the same way as described in **(A)**. **(C)** Real-time PCR analysis of *Nos2* and *Tnfa* mRNA in BMDMs pretreated with CsA (5 μM) or DMSO for 2 h, followed by treatment with IFN-γ (20 ng/mL) for various times. **(D)** Western blotting analysis of iNOS protein in BMDMs treated in the same way as described in **(C)**. **(E)** Real-time PCR analysis of *Arg1* and *Tgfb1* mRNA in BMDMs pretreated with CsA (5 μM) or DMSO for 2 h, followed by treatment with IL-4 (20 ng/mL) for various times. **(F)** Western blotting analysis of Arg1 protein in BMDMs treated in the same way as described in **(E)**. **(G)** Flow cytometry analysis of BMDMs pretreated in the same way as described in **(A)**, followed by infection with IAV (MOI = 1) for 24 h. F4/80+ cells representing macrophages (M0), CD80+ cells representing M1 macrophages, and CD206+ cells representing M2 macrophages. **(H)** Flow cytometry analysis of BMDMs pretreated in the same way as described in **(C)**, followed by treatment with IFN-γ (20 ng/mL) for 48 h. **(I)** Flow cytometry analysis of BMDMs pretreated in the same way as described in **(E)**, followed by treatment with IL-4 (20 ng/mL) for 48 h. Data are representative of at least three independent experiments. Data are presented as the mean ± SD. ns, not significant; *p < 0.05; **p < 0.01 (unpaired, two-tailed Student’s t-test).

Upon encountering different stimuli, macrophages polarize into M1 macrophages or M2 macrophages, which release a vastly different array of cytokines and chemokines that can either promote or inhibit inflammation (19, 23). We hypothesized that CsA might suppress IAV-triggered inflammatory responses by regulating macrophages polarization. Real-time PCR and western blotting assays were performed to investigate the expression of iNOS (a marker of M1 macrophages) and Arg1 (a marker of M2 macrophages) in IAV-infected BMDMs. As expected, CsA significantly reduced the production of iNOS and enhanced the production of Arg1 **(** **Figures 2A, B** **)**. Similar effect of CsA on the mRNA and protein expression levels of iNOS and Arg1, as well as the mRNA expression levels of *Tnfa* and *Tgfb1* were observed in IFN-γ-stimulated BMDMs **(** **Figures 2C, D** **)** and IL4-stimulated BMDMs **(** **Figures 2E, F** **)**. These results indicate that CsA is able to regulate macrophages polarization. The flow cytometry was performed to further define the phonotype of BMDMs during IAV infection. As shown in **Figure 2G**, CsA downregulated the proportion of M1 macrophages and upregulated the proportion of M2 macrophages in IAV-infected BMDMs. Moreover, CsA inhibited IFN-γ-induced M1 polarization **(** **Figure 2H** **)** and promoted IL-4-induced M2 polarization in BMDMs **(** **Figure 2I** **)**. These results suggest that CsA inhibits M1 polarization and promotes M2 polarization in BMDMs.

**Figure 3:**
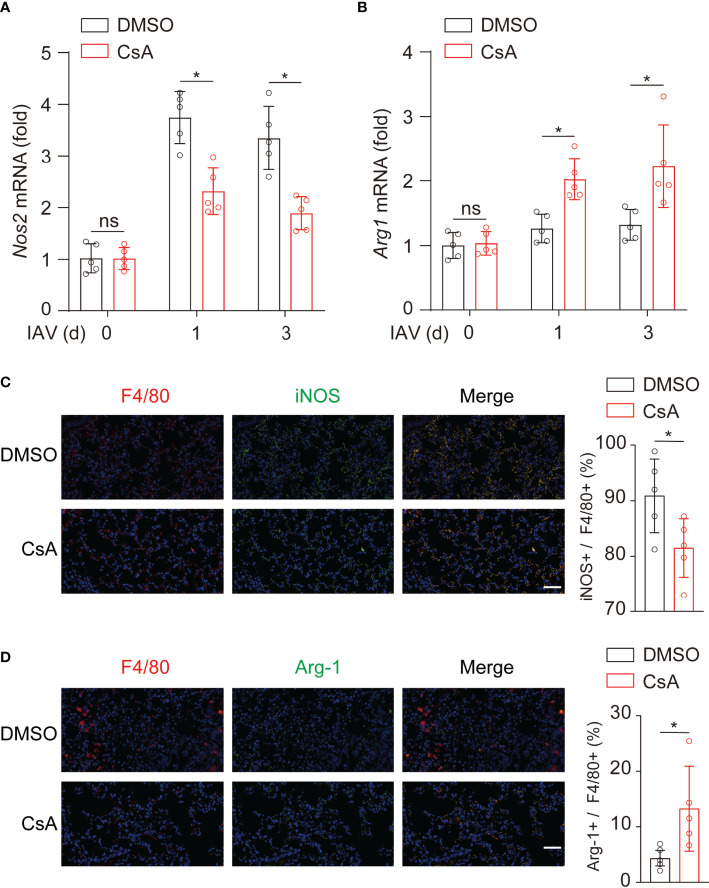
CsA regulates macrophages polarization upon IAV infection in mice. **(A, B)** Real-time PCR analysis of *Nos2* and *Arg1* mRNA in lungs from the mice in **Figure 1A** (n = 5). **(C)** Immunostaining of F4/80+ and iNOS+ macrophages in lungs of the mice in **Figure 1A** at day 3 post infection (n = 5). Scale bar, 50 μm. **(D)** Immunostaining of F4/80+ and Arg1+ macrophages in lungs of the mice in **Figure 1A** at day 3 post infection (n = 5). Scale bar, 50 μm. Data are representative of at least three independent experiments. Data are presented as the mean ± SD. ns, not significant; *p < 0.05 (unpaired, two-tailed Student’s t-test).

To determine whether CsA regulated macrophages polarization in mice, the expression levels of iNOS and Arg-1 were further examined in lungs from IAV-infected mice. During IAV infection, the mRNA levels of iNOS **(** **Figure 3A** **)** were decreased while those of Arg-1 **(** **Figure 3B** **)** were upregulated in CsA-treated mice. In addition, the immunostaining assays were performed. After IAV infection, the F4/80+ and iNOS+ cells were significantly decreased in lungs of CsA-pretreated mice **(** **Figure 3C** **)**. In contrast, the F4/80+ and Arg1+ cells were increased in lungs of CsA-pretreated mice **(** **Figure 3D** **)**. Collectively, CsA regulates macrophages polarization in both cell and mouse models.

### CsA Regulates Macrophages Polarization-Associated IFN-γ/STAT1 and IL4/STAT6 Signaling Pathways

**Figure 4:**
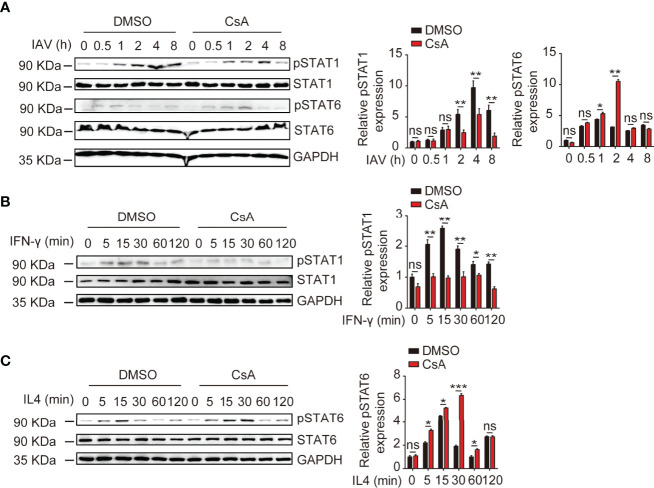
CsA regulates IFN-γ/STAT1 and IL-4/STAT6 signaling pathways. **(A)** Western blotting analysis of the indicated proteins in BMDMs pretreated with CsA (5 μM) or DMSO for 2 h before infection with IAV (MOI = 1) for various times (left). The relative expression levels of pSTAT1 and pSTAT6 were quantified. **(B)** Western blotting analysis of the indicated proteins in BMDMs pretreated with CsA (5 μM) or DMSO for 2 h, followed by treatment with IFN-γ (20 ng/mL) for various times (left). The relative expression levels of pSTAT1 were quantified. **(C)** Western blotting analysis of the indicated proteins in BMDMs pretreated with CsA (5 μM) or DMSO for 2 h, followed by treatment with IL-4 (20 ng/mL) for various times (left). The relative expression levels of pSTAT6 were quantified. Data are representative of at least three independent experiments. Data shown are the mean ± SD. ns, not significant; *p < 0.05; **p <0.01; ***p < 0.001 (unpaired, two-tailed Student’s t-test).

It is well established that the IFN-γ/STAT1 pathway is critical for M1 polarization and the IL4/STAT6 pathway is necessary for M2 polarization (24). Thus, we examined the effects of CsA on STAT1 and STAT6 phosphorylation upon IAV, IFN-γ, or IL4 stimulation. The immunoblotting results showed that CsA inhibited STAT1 phosphorylation and promoted STAT6 phosphorylation in IAV-infected BMDMs **(** **Figure 4A** **)**. Furthermore, after stimulation with IFN-γ or IL4, the lower level of pSTAT1 and higher level of pSTAT6 were found in CsA-treated BMDMs **(** **Figures 4B, C** **)**. Taken together, these results indicate that CsA regulates IFN-γ/STAT1 and IL4/STAT6 signaling pathways to influence macrophages polarization.

### CsA Regulates BMDMs Polarization by Targeting CypA

**Figure 5:**
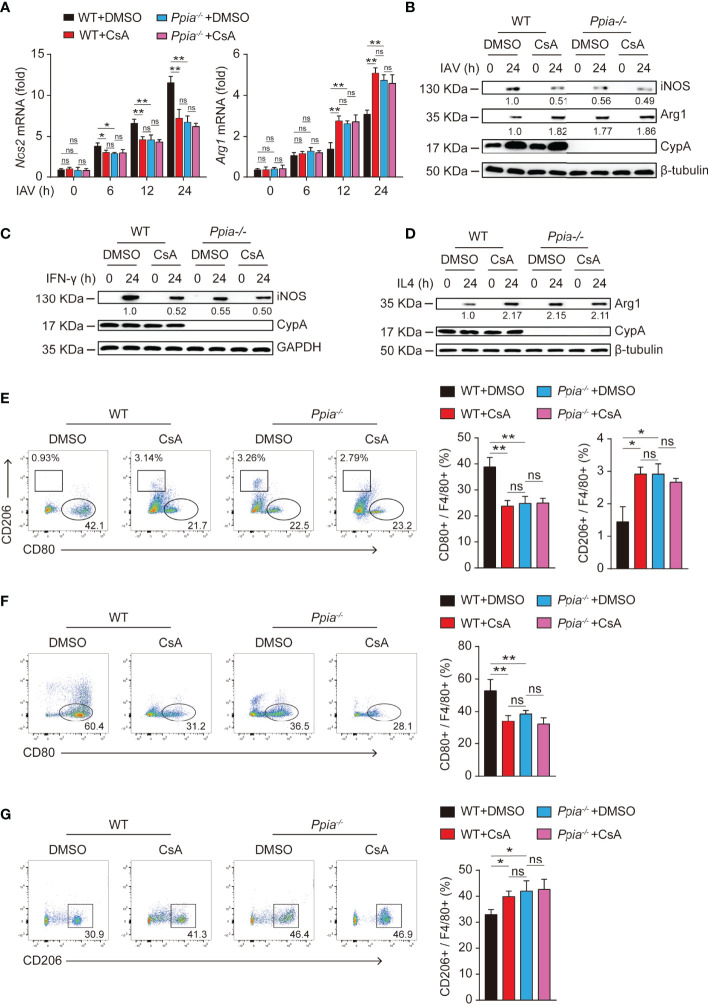
CsA regulates the polarization of BMDMs by targeting CypA. **(A)** Real-time PCR analysis of *Nos2* and *Arg1* mRNA in WT and *Ppia^-/-^* BMDMs pretreated with CsA (5 μM) or DMSO for 2 h before infected with IAV (MOI = 1) for various times. **(B)** Western blotting analysis of iNOS and Arg1 expression in WT and *Ppia^-/-^* BMDMs pretreated with CsA (5 μM) or DMSO for 2 h before infected with IAV (MOI = 1) for various times. **(C)** Western blotting analysis of iNOS expression in WT and *Ppia^-/-^* BMDMs pretreated with CsA (5 μM) or DMSO for 2 h before treated with IFN-γ (20 ng/mL) for various times. **(D)** Western blotting analysis of Arg1 expression in WT and *Ppia^-/-^* BMDMs pretreated with CsA (5 μM) or DMSO for 2 h before treated with IL4 (20 ng/mL) for various times. **(E)** Flow cytometry analysis of WT and *Ppia^-/-^* BMDMs pretreated with CsA (5 μM) or DMSO for 2 h before infected with IAV (MOI = 1) for 24 h. **(F)** Flow cytometry analysis of WT and *Ppia^-/-^* BMDMs pretreated with CsA (5 μM) or DMSO for 2 h before treated with IFN-γ (20 ng/mL) for 48 h. **(G)** Flow cytometry analysis of WT and *Ppia^-/-^* BMDMs pretreated with CsA (5 μM) or DMSO for 2 h before treated with IL-4 (20 ng/mL) for 48 h. Data are representative of three independent experiments. Data are presented as the mean ± SD. ns, not significant; *p < 0.05 **p < 0.01 (unpaired, two-tailed Student’s t-test).

It is well known that CypA is the main intracellular receptor of CsA, and many biological roles of CypA can be suppressed by CsA (25, 26). Therefore, we further investigated whether CsA involvement on macrophages polarization requires CypA. When CypA is knocked out, iNOS expression was decreased and Arg-1 expression was increased in IAV-infected **(** **Figures 5A, B** **)** or cytokines-stimulated **(** **Figures 5C, D** **)** BMDMs. Similar results were also found when WT BMDMs were pretreated with CsA. At the same time, the effect of CsA was diminished in *Ppia-*knockout (*Ppia^-/-^*) BMDMs. Moreover, the results of flow cytometry showed that CypA deficiency or CsA treatment led to the decreased proportion of M1 and the increased proportion of M2 in IAV-infected **(** **Figure 5E** **)** or cytokines-stimulated **(** **Figures 5F, G** **)** BMDMs, and CsA had no influence on macrophages polarization in CypA-deficient BMDMs. We also investigated the effect of CypA on the phosphorylation of STAT1 and STAT6. The lower phosphorylation levels of STAT1 and higher phosphorylation levels of STAT6 were observed in *Ppia^-/-^* BMDMs and CsA-treated WT BMDMs compared with those in WT BMDMs after IAV infection. Meanwhile, CsA had no effect on the phosphorylation of STAT1 and STAT6 in CypA-deficient BMDMs **(** **Supplementary Figure 1A** **)**. Similar results were observed in IFN-γ-stimulated BMDMs **(** **Supplementary Figure 1B** **)** and IL4-stimulated BMDMs **(** **Supplementary Figure 1C** **)**. Collectively, CypA deficiency decreases the polarization of macrophages into pro-inflammatory M1 phenotype and increases the polarization of macrophages into anti-inflammatory M2 phenotype just as CsA does and CsA regulates BMDMs polarization by targeting CypA to block its isomerase activity.

### CsA Targets CypA and Inhibits IAV-Induced Inflammatory Cytokines Production and Lung Injury

**Figure 6:**
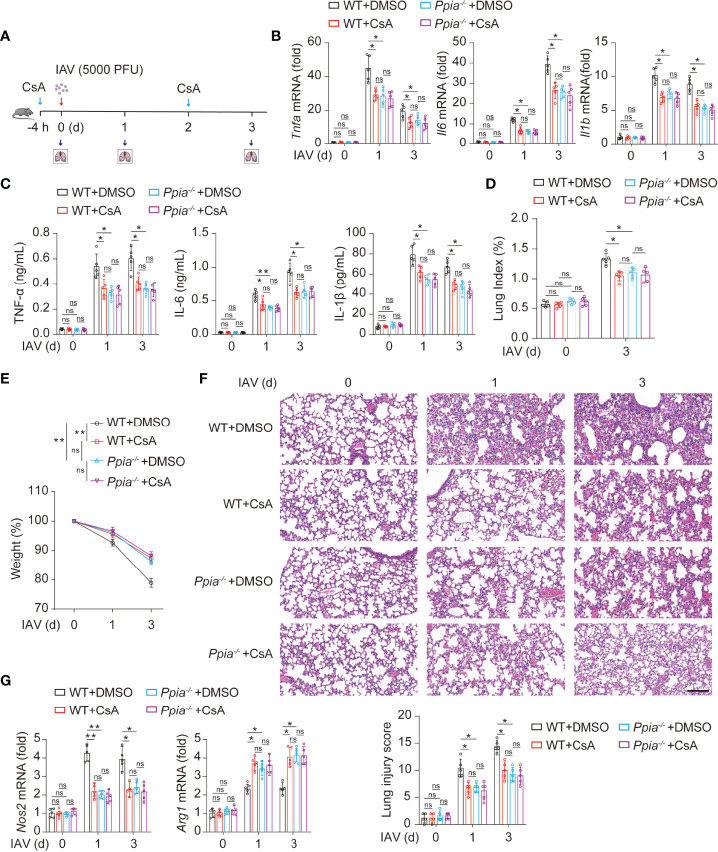
CsA targeted CypA and inhibited IAV-induced inflammatory cytokines production and lung injury in mice. **(A)** The WT and *Ppia^-/-^* Mice were treated in the same way as described in Fig. 1A (n = 5). **(B)** Real-time PCR analysis of *Tnfa*, *Il6*, and *Il1b* mRNA in lungs from WT or *Ppia^-/-^* mice. **(C)** ELISA analysis of TNF-α, IL-6, and IL-1β in BALF from mice. **(D)** The Lung index (100× lung/body weight) was calculated at day 0 and 3 post infection. **(E)** The body weight of mice was monitored at 0, 1 and 3 d after IAV infection. **(F)** Hematoxylin and eosin (H & E) staining of mouse lungs (top). The severity of the lung injury was analyzed in a blinded manner (bottom). Scale bar, 100 μm. **(G)** Real-time PCR analysis of *Nos2* and *Arg1* mRNA in mouse lungs. Data are representative of three independent experiments. Data are presented as the mean ± SD. ns, not significant; *p < 0.05; **p < 0.01 (unpaired, two-tailed Student’s t-test).

We further investigate whether the role of CsA in inflammatory responses is dependent on CypA in IAV-induced mice. We used the influenza A/WSN/33 model described on **Figure 1A** **(** **Figure 6A** **)**. The results of real-time PCR and ELISA assays revealed that the mRNA and protein levels of inflammatory cytokines in lungs of *Ppia^-/-^* mice or CsA-treated WT mice were lower than those in lungs of WT mice, and the effect of CsA was diminished in *Ppia^-/-^* mice **(** **Figures 6B, C** **)**. The pathological indices also indicated that the effect of CypA deficiency was similar with that of CsA treatment, and CsA no longer attenuated the pathological injury in *Ppia^-/-^* mice **(** **Figures 6D** **–F** **)**. Moreover, the effect of CypA deficiency on *Nos2* and *Arg1* transcriptional levels in mouse lungs were similar with that of CsA treatment, and CsA no longer had effect on *Nos2* and *Arg1* expression in *Ppia^-/-^* mice **(** **Figure 6G** **)**, suggesting that CypA played an important role in regulating macrophages polarization. Taken together, these results indicate that CsA suppresses IAV-induced inflammatory responses by targeting CypA.

## Discussion

**Figure 7:**
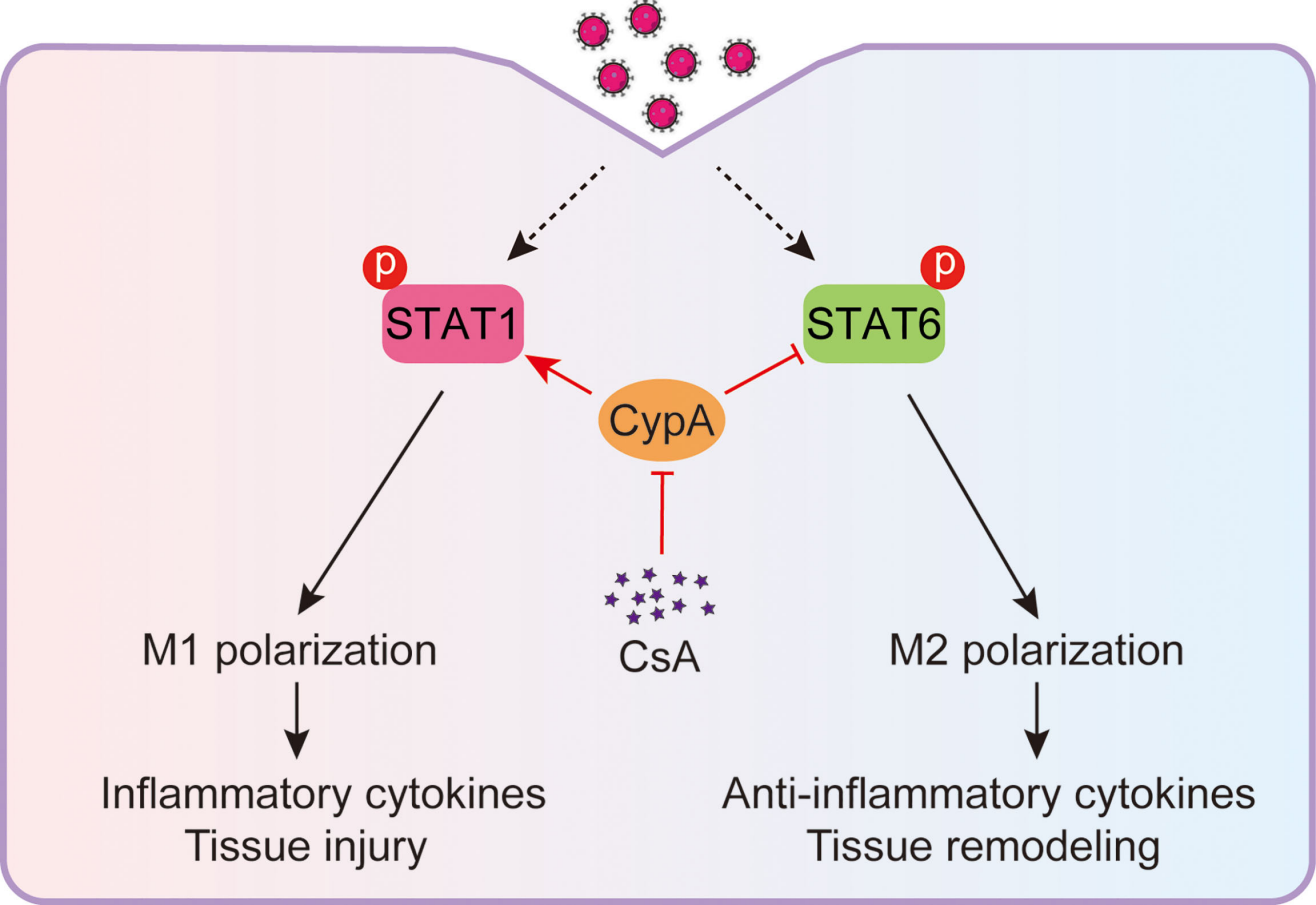
Model: CsA attenuates IAV-induced macrophages polarization and inflammatory responses by targeting CypA. CsA targets CypA to regulate IAV-triggered macrophages M1/M2 polarization by inhibiting STAT1 phosphorylation and promoting STAT6 phosphorylation, thereby suppressing inflammatory cytokines production and tissue injury.

IAV-induced acute inflammatory responses are a major threat to human health (27, 28), especially for the elderly and children (29). Respiratory epithelial cells are the primary target cells for IAV infection, IAV also spreads to neighboring macrophages after replication in the lungs (30, 31). The IAV-infected macrophages rapidly activate and produce inflammatory cytokines. These cytokines recruit more immune cells into pulmonary tissues and cause tissue injury (32). Therefore, macrophages play a central role in the inflammatory responses during the early stage of IAV infection (33). It has been reported that the immunosuppressive drug CsA is helpful to relieve acute inflammatory diseases (16). In the present study, we discover that CsA targets CypA and inhibits STAT1-mediated M1 macrophages polarization and promotes STAT6-mediated M2 macrophages polarization (**Figure 7**), providing a mechanism of CsA in suppressing inflammatory responses and tissue injury.

Based on the immunosuppressive effects on T lymphocytes, the early studies focus on solid organ transplantation and some autoimmune diseases (11, 34). However, the increasing evidence supports the role of CsA in regulating innate immune cells. CsA inhibits the expression of pro-inflammatory cytokines in LPS-stimulated macrophages (35), and suppresses the inflammatory responses of neutrophils upon phorbol-12-myristate-13-acetate (PMA), ionomycin, or IL-8 stimulation (36, 37). In some models, CsA inhibits the release of damage-associated molecular patterns and acts on the mitochondrial apoptotic pathway in acute inflammation (38). Here we show that CsA attenuates IAV-induced inflammatory responses by suppressing M1 macrophages polarization and promoting M2 macrophages polarization. This supports previously published data that the inhibition of M1 macrophages polarization results in reduced lung injury and mortality of mice (18, 39), which is one of the strategies for the treatment of inflammation-induced lung injury.

A complex network of cytokines, signaling molecules, transcription factors, and epigenetic mechanisms underlies the macrophage polarization (40). The STAT signaling pathways are activated by IFNs or TLR4 to skew macrophage toward M1 phenotype (*via* STAT1), or by IL-4 or IL-13 to skew toward M2 phenotype (*via* STAT6) (41). We demonstrate that CsA is able to regulate the polarization of macrophages by IFN-γ/STAT1 and IL4/STAT6 signaling pathways. In addition, canonical NF-κB activation promotes M1 macrophages polarization (42) However, induction of p50 NF-κB homodimers inhibits STAT1 activity and promotes M2 macrophages polarization *in vivo* (43). Many host factors have been demonstrated to play variety of roles in macrophages polarization, such as the members of SOCS family. IL-4 and IFN-γ facilitate the expression of SOCS1 and SOCS3, which in turn suppress the phosphorylation of STATs (44). We discover that CypA also takes part in regulating macrophages polarization. Whether other factors are involved in CsA-regulated macrophages polarization are unknown, which remains to be further studied.

In this study, we observe that CypA deficiency and CsA treatment have similar effects on macrophages polarization and inflammatory responses, and CsA no longer has these effects when CypA is absent, suggesting that CsA plays these roles by targeting CypA. CypA plays important roles in pathogen infection and host immunity. CypA is an IAV inducible host factor that promotes the coinfection of IAV and group A *Streptococcus* (GAS) by positively regulating the expression of integrin α5 and actin rearrangement *via* the FAK/Akt signaling pathway. In contrast, CsA treatment significantly inhibited IAV-GAS coinfection by negatively regulating the FAK/Akt signaling pathway (26). In addition, CypA regulates the ubiquitination of retinoic acid-inducible gene I (RIG-I) and mitochondrial antiviral-signaling (MAVS), promoting the production of type I interferon, thereby facilitating RIG-I-mediated antiviral, innate immune responses (45). How CypA regulate macrophages polarization and inflammatory responses is interesting and worthy of further study.

In conclusion, this study reveals a previously unpublished mechanism of CsA in regulating IAV-induced acute inflammatory responses. CsA inhibits the polarization of M1 and facilitates M2 polarization upon IAV infection by targeting CypA, and thereby suppressing the production of inflammatory cytokines and tissue injury. These data expand the knowledge on the biological functions of CsA in the field of acute inflammatory responses and provide a mechanism of the phenomenon that the use of CsA alleviates inflammatory responses.

## Materials And Methods

### Mice

Female C57BL/6 mice (6-8 weeks old, 18-22 g) were purchased from Vital River Laboratory Animal Center (Beijing, China). *Ppia*-knockout (*Ppia^-/-^*) 129 mice were purchased from Jackson Laboratory and backcrossed for at least six generations to C57BL/6 mice. All of the animals were maintained in a barrier facility with free access to food and water.

### Cells and Viruses

The Madin–Darby canine kidney cells (MDCK, ATCC CRL-3216) was obtained from the American Type Culture Collection (ATCC). MDCK cell lines were tested for mycoplasma contamination using the MycoAlert Mycoplasma Detection Kit (Lonza, Switzerland) and maintained in Dulbecco’s modified Eagle’s medium (DMEM, Gibco) supplemented with 10% (v/v) fetal bovine serum (FBS, Gibco), 100 U/mL penicillin and 100 μg/mL streptomycin at 37°C under a humidified atmosphere containing 5% CO_2_. BMCs (bone marrow cells) were isolated from aseptically dissected and flushed tibias and femurs of 6-8 weeks old C57BL/6 mice. BMCs were differentiated into bone marrow-derived macrophages (BMDMs) for 5-7 days in 1640 medium (Gibco) with 10% (v/v) FBS, 20 ng/ml macrophage-colony stimulating factor (M-CSF), 100 U/mL penicillin and 100 μg/mL streptomycin at 37°C under a humidified atmosphere containing 5% CO_2_.

Influenza A/WSN/33 (H1N1) was rescued from cDNA (46). Viruses were propagated in 9-day-old specific pathogen-free embryonic eggs or MDCK cells for 36–72 h at 37°C, then the allantoic fluid and supernatant was harvested, centrifuged, and stored at -80°C.

### Antibodies and CsA

For flow cytometry, F4/80-PE, CD80-Pacific Blue and CD206-APC antibodies were obtained from Thermo Fisher. For western blotting assays, the following antibodies were used: anti-pSTAT1 (1:1000, 9167S, CST), anti-STAT1 (1:500, sc-464, Santa Cruz), anti-pSTAT6 (1:1000, 56554S, CST), anti-STAT6 (1:1000, 5397S, CST), anti-GAPDH (1:2000, ab8245, Abcam). For immunostaining assays, the following antibodies were used: anti-F4/80 (1:100, 30325S, CST), anti-iNOS (1:100, 32027S, CST), anti-Arg1 (1:100, 93668S, CST). The CsA powder (S2286) was purchased from Selleck. For assays *in vitro*, the powder was dissolved with DMSO and 0.1% DMSO was the final concentration for cell culture. For assays *in vivo*, the powder was dissolved in a solution of 2% DMSO, 30% PEG300, and 5% Tween 80 in sterile ddH_2_O.

### Virus Titration

MDCK were inoculated with 10-fold serially diluted IAV and incubated at 37°C with 5% CO_2_ for 2 h. The supernatant was removed; cells were washed with PBS, and then overlaid with DMEM containing 0.6% low-melting-point agarose and 1 μg/mL L-(tosylamido-2-phenyl) ethyl chloromethyl ketone (TPCK)-treated trypsin. After incubation for 48-72 hour, the virus titers were determined by visible plaques.

### IAV Infection *In Vitro*

BMDMs were infected with IAV (MOI = 1) in DMEM containing 0.5 μg/mL TPCK-treated trypsin for 2 h. Subsequently, the supernatant was removed; cells were washed with PBS and overlaid with infection media (DMEM with 100 U/mL penicillin and 100 μg/mL streptomycin). Cells were collected at indicated time for the subsequent real-time PCR, flow cytometry and western blotting.

### The Polarization of BMDMs

BMDMs were polarized in 1640 medium containing IFN-γ (20 ng/mL) or IL4 (20 ng/mL), 100 U/mL penicillin and 100 μg/mL streptomycin for indicated time, followed by collected and used for real-time PCR, flow cytometry and western blotting.

### IAV Infection *In Vivo*

6–8 weeks old C57BL/6 mice were anesthetized and inoculated intranasally with IAV (5000 PFU) in 30 μL of PBS. After 1 or 3 day, the mice were sacrificed and the lungs were used in subsequent real-time PCR, lung index analysis, Hematoxylin and eosin (H & E) staining and immunostaining assays. Mouse body weights were monitored.

### RNA Extraction, cDNA Synthesis, and Real-Time PCR

Total RNA was extracted from BMDMs and lung tissue homogenate with TRIzol (Invitrogen) according to the manufacturer’s instructions. The cDNA was synthesized from 1-2 μg of total RNA using an oligo (dT) primer and M-MLV reverse transcriptase (Promega) according to the manufacturer’s instructions. The relative gene expression was analyzed by real-time PCR that using TB Green premix (TaKaRa). Primers used in this study: *Tnfa*, forward primer: 5′-CAT CTT CTC AAA ATT CGA GTG ACA A-3′, and reverse: 5′-TGG GAG TAG ACA AGG TAC AAC CC-3′; *Il6*, forward primer: 5′-CCA GAA ACC GCT ATG AAG TTC C-3′, and reverse: 5′-TTG TCA CCA GCA TCA GTC CC-3′; *Il1b*, forward primer: 5′-GTG GCT GTG GAG AAG CTG TG-3′, and reverse: 5′-GAA GGT CCA CGG GAA AGA CAC-3′; *Ccl2*, forward primer: 5′-CAG CCA GAT GCA ATC AAT GCC-3′, and reverse: 5′-TGG AAT CCT GAA CCC ACT TCT-3′; *Cxcl10*, forward primer: 5′-CCA AGT GCT GCC GTC ATT TTC T-3′, and reverse: 5′-TTC CCT ATG GCC CTC ATT CTC A-3′; *Il4*, forward primer: 5′-CCC CAG CTA GTT GTC ATC CTG-3′, and reverse: 5′-CAA GTG ATT TTT GTC GCA TCC G-3′; *Il10*, forward primer: 5′-CTT TAA GGG TTA CTT GGG TTG CC-3′, and reverse: 5′-TCC TGA GGG TCT TCA GCT TCT CA-3′; *Tgfb1*, forward primer: 5′-TTA GGA AGG ACC TGG GTT GGA-3′, and reverse: 5′-CCG GGT TGT GTT GGT TGT AGA-3′; *Nos2*, forward primer: 5′-GGC AGC CTG TGA GAC CTT TG-3′, and reverse: 5′-GCA TTG GAA GTG AAG CGT TTC-3′; *Arg1*, forward primer: 5′-CTC CAA GCC AAA GTC CTT AGA G-3′, and reverse: 5′-AGG AGC TGT CAT TAG GGA CAT C-3′; *Gapdh*, forward primer: 5′-GGT GGT CTC CTC TGA CTT CAA CA-3′, and reverse: 5′-GTT GCT GTA GCC AAA TTC GTT GT-3′. The Ct values generated from ABI 7500 and were analyzed by 2^−ΔΔCt^ method. The expression of target genes was normalized to Gapdh.

### Western Blotting

Cells were lysed in lysis buffer (150 mM NaCl, 20 mM Hepes, 1 mM EDTA, 1% TritonX-100, 10% glycerin and protease or phosphatase inhibitors cocktail). The total protein was quantified by BCA protein assay kit (Thermo Fisher) and the same mass of protein samples with 5×Loading buffer were loaded on 12% sodium dodecyl sulfate (SDS)-polyacrylamide gel, and then electroblotted onto a 0.45 μm PVDF membrane. The membranes were blocked by TBST (10 mM Tris-HCl, 150 mM NaCl, 0.1% Tween 20, pH 8.0) with 5% (w/v) non-fat dry milk and 1% (w/v) BSA for 1.5 h, followed by incubated with primary antibodies at room temperature for 2 h and washed by TBST. Subsequently, the HRP conjugated specific secondary antibodies was used to bind target primary antibodies for 1 h. After washing by TBST, the target bands were visible by using the ECL western blotting substrate. The expression of target protein was normalized to GAPDH.

### Enzyme Linked Immunosorbent Assay

Bronchoalveolar lavage fluid (BALF) was obtained from the lungs of mice by washing with 1 mL PBS and collected after centrifugation. The concentration of TNF-α (MTA00B, R&D systems), IL6 (M6000B, R&D systems) and IL1β (MLB00C, R&D systems) in BALF were measured by ELISA according to the manufacturer’s instructions.

### Flow Cytometry

FACS analyses were done on BD FACSAria. Data were analyzed with the FlowJo software (Tree Star Inc.).

### Lung Injury Severity Scoring

The severity of the lung injury was analyzed in a blinded manner with the grader unaware of the concrete group being reviewed. The lung histopathological changes were assessed by the four identifiable pathologic processes: (1) alveolar congestion, (2) hemorrhage, (3) leukocyte infiltration or aggregation of neutrophils in airspace or the vessel wall, and (4) thickness of the alveolar wall. The scores of 0 to 4 were defined to represent normal lungs, lower than 25%, 25–50%, 50–75%, and higher than 75% lung involvement, respectively.

## Statistical Analyses

Statistical analyses were done on Prism 9 (GraphPad) software and Microsoft Excel (2010). All data are presented as the mean values ± SD of at least three independent experiments. Comparisons between two groups were performed using the two-tailed Student’s t test. *P* < 0.05 was considered significant, with *p < 0.05; **p < 0.01; and ***p < 0.001.

## Data Availability Statement

The original contributions presented in the study are included in the article/**Supplementary Material**. Further inquiries can be directed to the corresponding authors.

## Ethics Statement

All mouse experiments procedures were reviewed and approved by the Research Ethics Committee of the Institute of Microbiology, Chinese Academy of Sciences and complied with the Beijing Laboratory Animal Welfare and Ethical Guidelines of the Beijing Administration Committee of Laboratory Animals.

## Author Contributions

LS initiated the project and supervised the project. LS and XB designed the experiments, analyzed the data, and wrote the paper. XB performed the experiments. WY, HL, YZ, and HZ helped with some experiments. WL and WF helped analyze the data and revised the manuscript. All authors contributed to the article and approved the submitted version.

## Funding

This work was supported by grants from the National Natural Science Foundation of China (31972657) and the Strategic Priority Research Program of the Chinese Academy of Sciences (XDB29010000).

## Conflict of Interest

The authors declare that the research was conducted in the absence of any commercial or financial relationships that could be construed as a potential conflict of interest.

## Publisher’s Note

All claims expressed in this article are solely those of the authors and do not necessarily represent those of their affiliated organizations, or those of the publisher, the editors and the reviewers. Any product that may be evaluated in this article, or claim that may be made by its manufacturer, is not guaranteed or endorsed by the publisher.

## Supplementary Material

Supplementary Figure 1 CsA regulates IFN-γ/STAT1 and IL-4/STAT6 signaling pathways by targeting CypA. **(A)** Western blotting analysis of the indicated proteins in WT and *Ppia^-/-^* bone marrow-derived macrophages (BMDMs) pretreated with CsA (5 μM) or DMSO for 2 h before infection with influenza A virus (MOI = 1) for various times (left). The relative expression levels of pSTAT1 and pSTAT6 were quantified. **(B)** Western blotting analysis of the indicated proteins in WT and *Ppia^-/-^* BMDMs pretreated with CsA (5 μM) or DMSO for 2 h before treated with IFN-γ (20 ng/mL) for various times (left). The relative expression levels of pSTAT1 were quantified. **(C)** Western blotting analysis of the indicated proteins in WT and *Ppia^-/-^* BMDMs pretreated with CsA (5 μM) or DMSO for 2 h before treated with IL-4 (20 ng/mL) for various times (left). The relative expression levels of pSTAT6 were quantified. Data are representative of three independent experiments. Data are presented as the mean ± SD. ns, not significant; **p* < 0.05 (unpaired, two-tailed Student’s t-test).
