# Supplementary figures and images for: Cyclosporine A Regulates Influenza A Virus-induced Macrophages Polarization and Inflammatory Responses by Targeting Cyclophilin A
Source: Front Immunol. 2022 May 25;13:861292. doi: 10.3389/fimmu.2022.861292 (PMC9174699; doi:10.3389/fimmu.2022.861292)

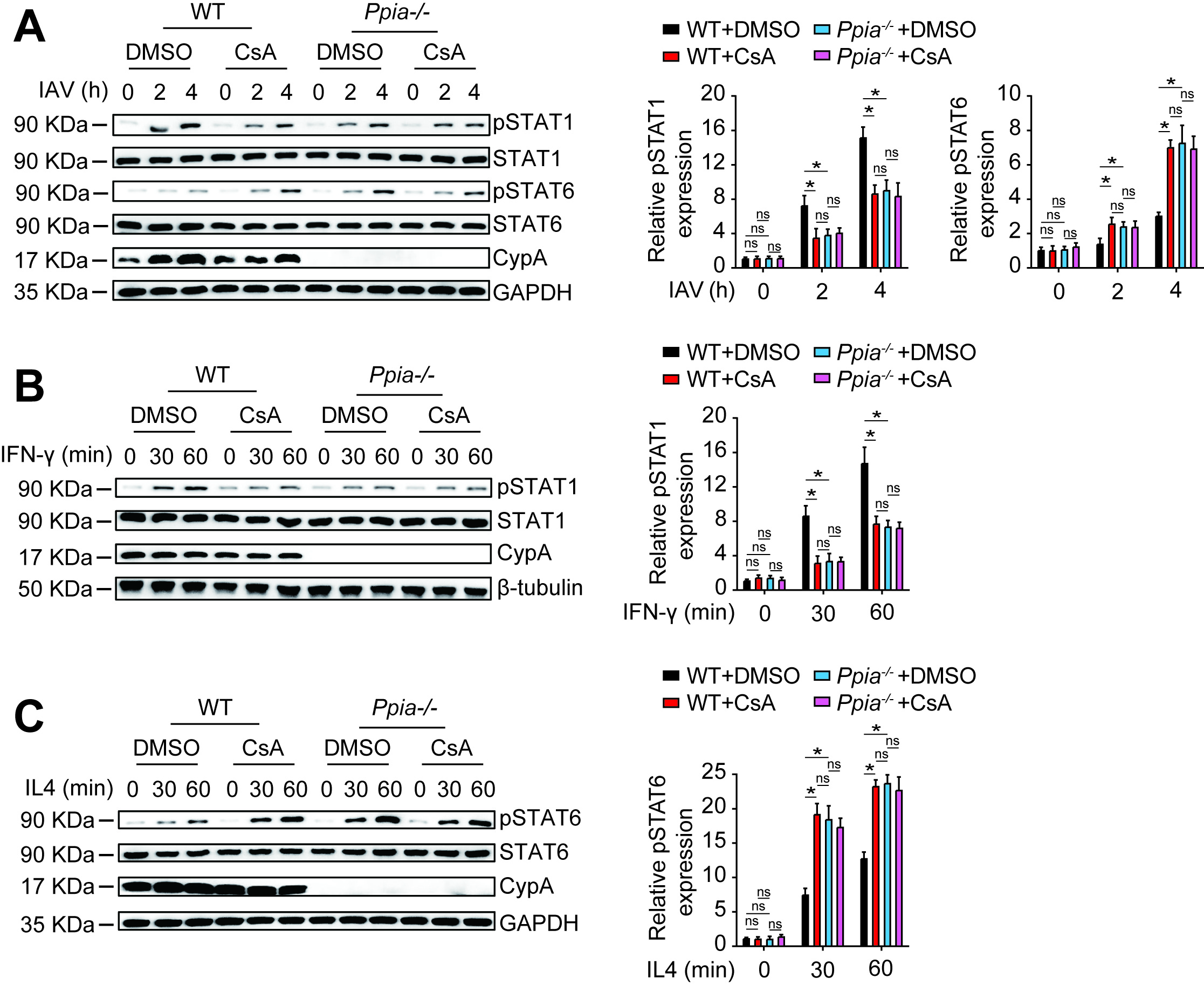

Supplement: Supplementary Figure 1 — CsA regulates IFN-γ/STAT1 and IL-4/STAT6 signaling pathways by targeting CypA. (A) Western blotting analysis of the indicated proteins in WT and Ppia-/- bone marrow-derived macrophages (BMDMs) pretreated with CsA (5 μM) or DMSO for 2 h before infection with influenza A virus (MOI = 1) for various times (left). The relative expression levels of pSTAT1 and pSTAT6 were quantified. (B) Western blotting analysis of the indicated proteins in WT and Ppia-/- BMDMs pretreated with CsA (5 μM) or DMSO for 2 h before treated with IFN-γ (20 ng/mL) for various times (left). The relative expression levels of pSTAT1 were quantified. (C) Western blotting analysis of the indicated proteins in WT and Ppia-/- BMDMs pretreated with CsA (5 μM) or DMSO for 2 h before treated with IL-4 (20 ng/mL) for various times (left). The relative expression levels of pSTAT6 were quantified. Data are representative of three independent experiments. Data are presented as the mean ± SD. ns, not significant; *p < 0.05 (unpaired, two-tailed Student’s t-test). [file Image_1.jpeg]
